# Quorum-Sensing Master Regulator VfmE Is a c-di-GMP Effector That Controls Pectate Lyase Production in the Phytopathogen Dickeya dadantii

**DOI:** 10.1128/spectrum.01805-21

**Published:** 2022-03-30

**Authors:** Biswarup Banerjee, Quan Zeng, Manda Yu, Brian Y. Hsueh, Christopher M. Waters, Ching-Hong Yang

**Affiliations:** a Department of Biological Sciences, University of Wisconsin-Milwaukee, Milwaukee, Wisconsin, USA; b Department of Plant Pathology and Ecology, The Connecticut Agricultural Experiment Station, New Haven, Connecticut, USA; c Department of Microbiology and Molecular Genetics, Michigan State University, East Lansing, Michigan, USA; University of Minnesota

**Keywords:** quorum sensing, c-di-GMP, VfmE, *Dickeya dadantii*, pectate lyase

## Abstract

Dickeya dadantii is a phytopathogenic bacterium that causes diseases on a wide range of host plants. The pathogen secretes pectate lyases (Pel) through the type II secretion system (T2SS) that degrades the cell wall in host plants. The virulence of *D. dadantii* is controlled by the second messenger cyclic diguanylate monophosphate (c-di-GMP), and the homeostasis of c-di-GMP is maintained by a number of diguanylate cyclases and phosphodiesterases. Deletion of a phosphodiesterase *ecpC* repressed *pelD* transcription, and such repression can be suppressed by an additional deletion in *vfmE*. VfmE is an AraC type of transcriptional regulator in the Vfm quorum-sensing system. Our results suggest that VfmE is a c-di-GMP effector that functions as an activator of *pel* at low c-di-GMP concentrations and a repressor of *pel* at high c-di-GMP concentrations through regulation of the transcriptional activator SlyA. Multiple sequence alignment with known c-di-GMP effectors identified an RWIWR motif in VfmE that we demonstrate is required for the c-di-GMP binding. Mutation of R93D in the RxxxR motif eliminates the c-di-GMP-related phenotypes in Pel activity. Our results show that VfmE is not only a quorum-sensing regulator but also a c-di-GMP effector, suggesting that *D. dadantii* integrates the c-di-GMP signaling network with the Vfm quorum-sensing pathway during environmental adaptation.

**IMPORTANCE** How bacteria integrate environmental cues from multiple sources to appropriately regulate adaptive phenotypes is a central question in microbiology. In Dickeya dadantii, the quorum-sensing regulator VfmE controls the key virulence factor pectate lyase (Pel). Here, we demonstrate that VfmE also binds to c-di-GMP, resulting in VfmE functioning as an activator of *pel* at low c-di-GMP concentrations and repressor of *pel* at high c-di-GMP concentrations. The RWIWR motif in VfmE is required for c-di-GMP binding, and mutation of the motif in the mutant R93D eliminates the c-di-GMP-related phenotypes in Pel activity. We propose that VfmE is an important mediator to integrate quorum-sensing signals with c-di-GMP to collectively regulate *D. dadantii* pathogenesis.

## INTRODUCTION

Dickeya dadantii, a bacterium of the *Enterobacteriaceae* family, is a pectinolytic plant pathogen causing soft rot, wilt, and blight diseases on a wide range of important crop species, such as tomato and potato (1). *D. dadantii* causes infection in the apoplast of plants tissue. Large maceration areas on plant leaves and tissues appear due to their degradation by the plant cell wall-degrading enzymes (CWDEs), such as pectate lyases (Pel), proteases, cellulases, and polygalacturonases (2). *D. dadantii* secretes Pels into the host via the type II secretion system (T2SS), and it encodes several Pel genes—*pelA*, *pelB*, *pelC*, *pelD*, and *pelZ*. Among these pectate lyases, PelD is regarded as the major endo-pectate lyase enzyme that acts on low methoxylated homogalacturonan and catalyzes β-elimination at random galactosidic bonds (3, 4). Despite the identification of the virulence factors, how *D. dadantii* senses environmental and host signals and modulates the expression of such virulence factors remains unclear.

Bis-(3′-5′)-cyclic dimeric guanosine monophosphate (c-di-GMP) is a ubiquitous bacterial second-messenger molecule that regulates many cellular processes, such as CWDE production (5). Low intracellular c-di-GMP levels are associated with a planktonic lifestyle, while high c-di-GMP levels promote a sessile life cycle, such as biofilm formation. The turnover of intracellular c-di-GMP levels is subject to the regulation of two types of enzymes, diguanylate cyclase (DGC) and phosphodiesterase (PDE). DGCs are GGDEF domain-containing proteins that synthesize c-di-GMP from two GTP molecules. In contrast, PDEs are EAL or HD-GYP domain-containing proteins that hydrolyze c-di-GMP into 5′-phosphoguanylyl-(3′-5′)-guanosine (pGpG) or two molecules of guanosine monophosphates (GMP). c-di-GMP controls a wide variety of cellular functions through the binding of different effectors. The reported c-di-GMP effectors are structurally diverse, including PilZ-domain proteins, GGDEF domain proteins with an I-site, degenerate GGDEF or EAL domain proteins, and RNA riboswitches (6, 7). Our previous studies of *D. dadantii* reported that two PDEs, EcpC and EGcpB (or EcpB), and one DGC, GcpA, are involved in the regulation of pectate lyase production (4, 8–10). However, the effectors involved in the regulation of the CWDEs in *D. dadantii* remain unresolved.

Quorum sensing (QS) is the bacterial intercellular communication system that depends on small signaling molecules that are either perceived by the cells via transmembrane receptor proteins or membrane-permeable. QS is required for pathogenicity and host colonization in many plant-pathogenic bacteria (11). Two QS systems were reported previously in *D. dadantii*. The Exp (AHL-QS) system uses an N-acyl-homoserine lactone (AHL) autoinducer, which is ubiquitous among Gram-negative bacteria. The other quorum-sensing system is known as the Vfm QS system (virulence factor modulating cluster), which is conserved in *Dickeya* species (12). The *vfm* genes direct the synthesis of an extracellular signal and constitute a novel quorum-sensing system. The signal is perceived by the two-component system VfmI-VfmH, which activates the expression of an AraC regulatory protein, VfmE. VfmE activates both the transcription of the CWDE genes and the expression of the *vfm* operon. The *vfmP* is one of the QS signal-producing genes in the *vfm* operon that encodes amino acid-activating enzymes and displays similarities to the adenylation (A) domains of the nonribosomal peptide gramicidin synthetase (NRPS) (13).

In this study, we show that transposon insertion in *vfmE* suppresses the reduced *pelD* expression observed in the PDE deletion mutant Δ*ecpC*. We then show that VfmE regulates *pelD* through the transcriptional regulator SlyA, and the regulation is dependent on cellular c-di-GMP levels. Furthermore, we demonstrate that VfmE binds to c-di-GMP through a PilZ-like mechanism and is involved in the regulation of CWDE production. This study identified a previously characterized quorum-sensing regulator as a novel c-di-GMP effector and unveiled interacting pathways of quorum-sensing and secondary messenger c-di-GMP that together regulate virulence in *D*. *dadantii*.

## RESULTS

### Identification of a transposon mutant that alleviates the repression of Pel under high c-di-GMP conditions.

**FIG 1:**
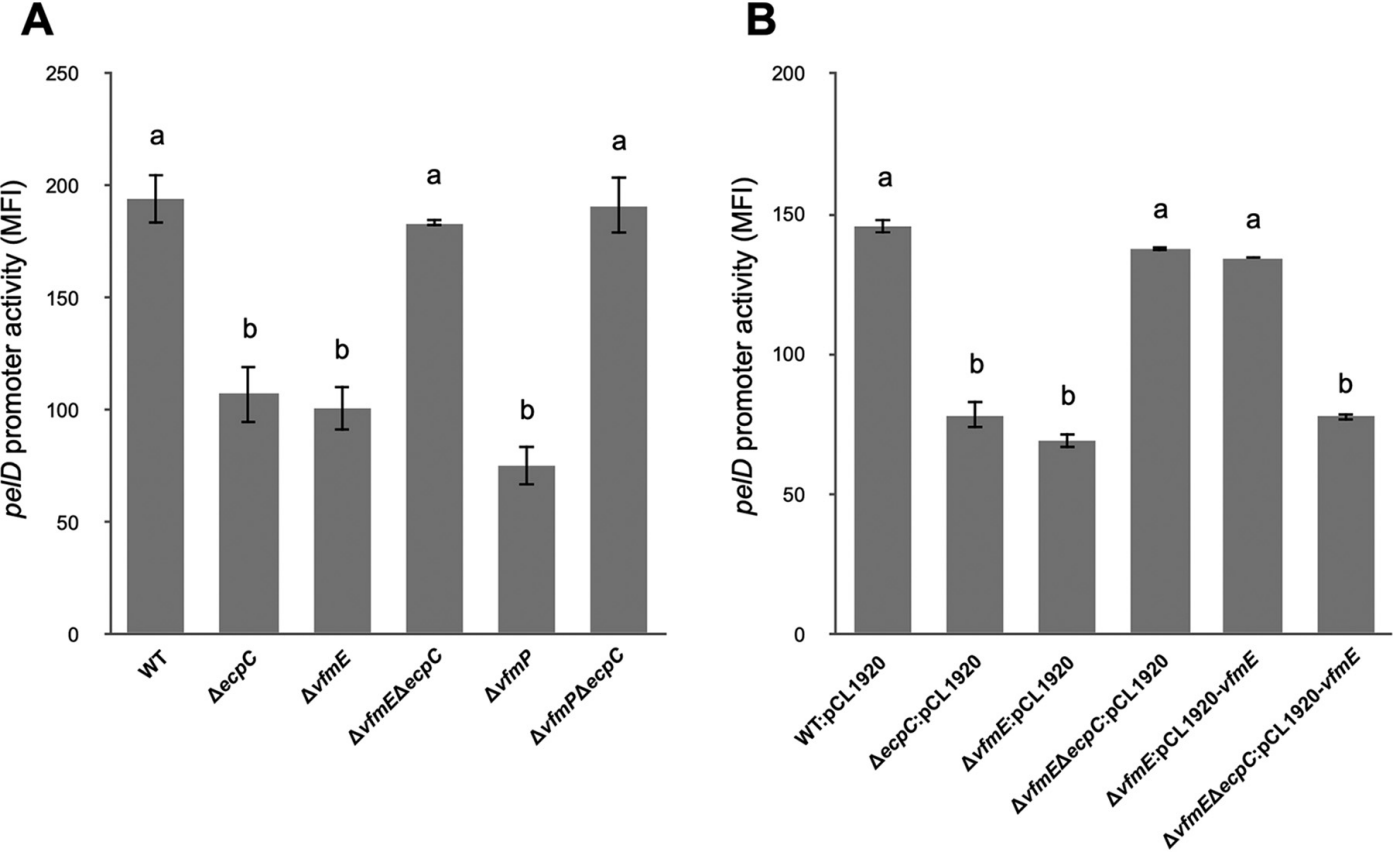
Deletion of *vfmE* and *vfmP* in the wild type (WT) decreases *pelD* promoter activity; on the other hand, deletion of both *vfmE* and *vfmP* under a high-c-di-GMP background (Δ*ecpC)* restores the *pelD* promoter activity to the WT level. The pPROBE-AT plasmid harboring the *pelD*-GFP promoter region was measured by flow cytometry. (A) *pelD* promoter activity in the wild type, Δ*ecpC*, Δ*vfmE*, Δ*vfmE* Δ*ecpC*, Δ*vfmP*, and Δ*vfmP* Δ*ecpC* was measured. (B) Complementation of *pelD* promoter activity in the wild type, Δ*ecpC*, Δ*vfmE*, and Δ*vfmE* Δ*ecpC* harboring low-copy-number empty pCL1920 plasmid (controls) and Δ*vfmE* and Δ*vfmE* Δ*ecpC* strains harboring pCL1920:*vfmE*. Mean fluorescence intensity (MFI) and average GFP fluorescence intensity of total bacterial cells was examined. The MFI is the log fluorescence intensity given by Cell Quest software (BD Biosciences, San Jose, CA). Values are representative of three experiments, and three replicates were used for each experiment. The lowercase letters represent different treatment groups with significant statistical differences, whereas treatments with no significant differences were assigned the same letters for 24 h in MM medium (*P* < 0.05) by one-way ANOVA.

Previous studies demonstrated that EcpC positively regulates the pectate lyase and transcription of *pelD* through c-di-GMP, yet the effector(s) that perceive the c-di-GMP signal and execute the transcriptional regulation remains unknown (4, 8). To identify the potential c-di-GMP effector involved in this regulation, the promoter of *pelD* was cloned to a *gfp* (green fluorescent protein) reporter plasmid and subsequently transferred into a Δ*ecpC* mutant. As EcpC is a phosphodiesterase that degrades c-di-GMP, the expression of *pelD* was significantly reduced in the *ecpC* mutant compared to the wild type (8) (Fig. 1A). Next, we constructed a transposon library in the Δ*ecpC* mutant carrying the *pelD* promoter*-gfp* fusion (*P_pelD_*::*gfp*) and screened for mutants with *pelD* transcription restored to the wild-type level by monitoring the GFP intensity. Out of 1,650 transposon mutants screened, 10 mutants restored *pelD* expression in Δ*ecpC* at a similar level as in the wild type (higher expression relative to Δ*ecpC*) (see the supplemental material). Two of the ten mutants contained transposon insertions in the open reading frames of *vfmE* (ABF-0016073) and *vfmP* (ABF-0019406). As these genes are quorum-sensing regulators, we further analyzed the functions of these genes in c-di-GMP sensing and virulence regulation.

**FIG 2:**
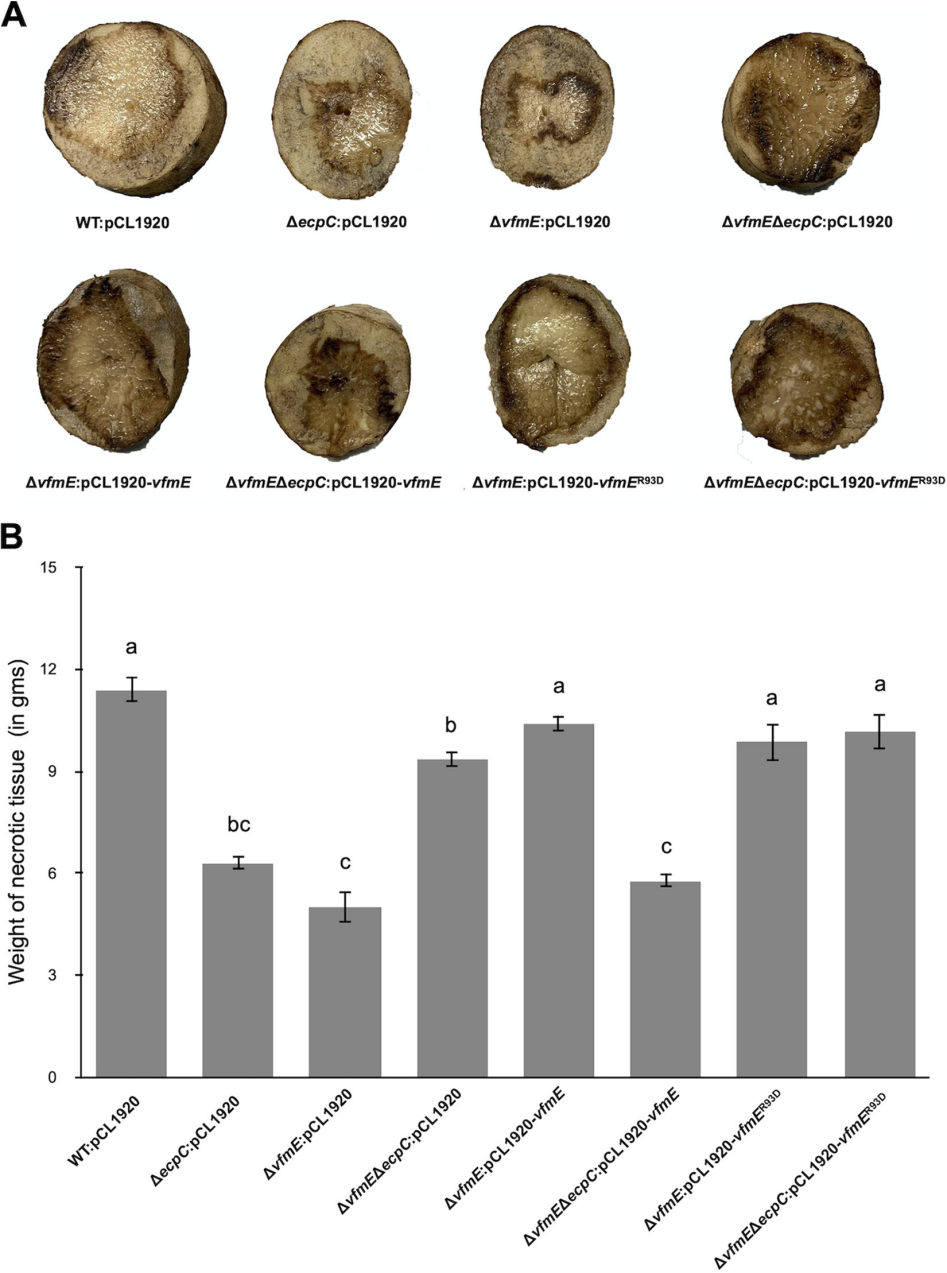
Deletion of *vfmE* in the wild type (WT) reduces the maceration area in potato; on the other hand, deletion of *vfmE* under a high-c-di-GMP background (Δ*ecpC)* restores the maceration area to the WT level. (A) The maceration areas caused by *D. dadantii* strains observed in potato host by wild type, Δ*ecpC*, Δ*vfmE*, and Δ*vfmE* Δ*ecpC* and the corresponding complementations with pCL1920 plasmid harboring *vfmE* and *vfmE*^R93D^ with its natural promoter. (B) The weights of the necrotic tissues measured in WT, Δ*ecpC*, Δ*vfmE*, and Δ*vfmE* Δ*ecpC* mutants and the complementation strains. Values are representative of two experiments, and three replicates were used for each experiment. The lowercase letters represent different treatment groups with significant statistical differences, whereas treatments with no significant differences were assigned the same letters for 24 h (*P* < 0.05) by one-way ANOVA.

To confirm our transposon mutagenesis data, we generated mutants by deleting *vfmE* or *vfmP* in the wild-type and Δ*ecpC* backgrounds and then tested the expression of *pelD* using a promoter fluorescence reporter. We observed reduced *pelD* expression in *vfmE* and *vfmP* single deletion mutants (Fig. 1A). This suggests that VfmE and VfmP positively regulate *pelD* gene expression, which is consistent with previous research on *vfm* regulons (13). Similar to the Δ*ecpC vfmE* transposon mutant, an increased expression of *pelD* was also observed in the Δ*vfmE* Δ*ecpC* and Δ*vfmP* Δ*ecpC* double deletion mutants (Fig. 1A). The altered *pelD* expression could be complemented in the Δ*vfmE* and Δ*vfmE* Δ*ecpC* mutants by introducing the *vfmE* gene with its native promoter cloned in pCL1920, a low-copy-number plasmid (Fig. 1B). Thus, although VfmE behaves as a positive regulator of PelD in WT *D. dadantii*, VfmE negatively regulates *pelD* expression in the Δ*ecpC* background, which reveals a complex role of VfmE in pectate lyase regulation. Since deletion of *vfmE* and double deletion of *vfmE* and *ecpC* showed opposite effects on *pelD* expression, we carried out virulence assays on potatoes. Consistent with the *pelD* expression results, Δ*ecpC* showed reduced necrotic tissue formation compared to the WT in potato (Fig. 2A and 2B). Reduced necrosis of potato tissues in the Δ*vfmE* single deletion mutant was observed, but more necrosis was observed in the Δ*vfmE* Δ*ecpC* double mutant that was on par with the WT strain. The virulence phenotypes observed in Δ*vfmE* and Δ*vfmE* Δ*ecpC* mutants could be complemented by the expression of the *vfmE* gene in *trans* (Fig. 2A and B). The above-described data show that Δ*vfmE* and Δ*ecpC* reduce the Pel activity, but the Δ*vfmE* Δ*ecpC* double mutant restores the Pel production to the WT level. In addition, in *trans* expression of *vfmE* can complement the phenotypes under a low c-di-GMP background but fails to complement the phenotypes under a high c-di-GMP background.

**FIG 3:**
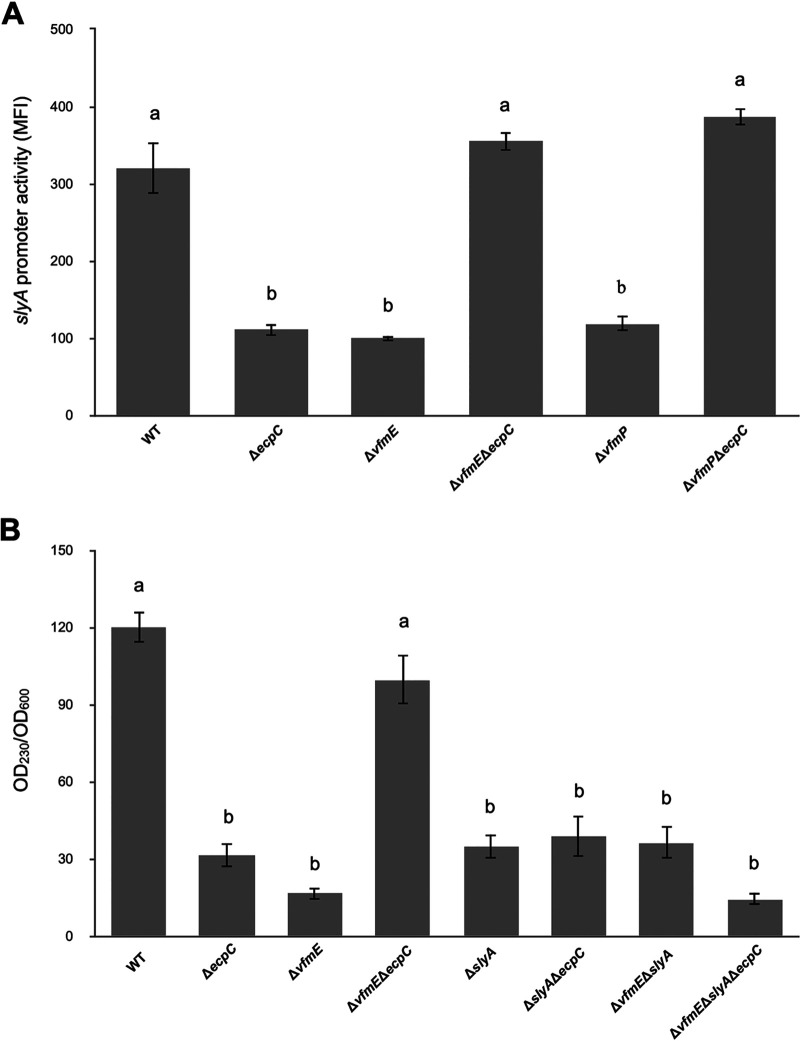
Regulation of Pel by VfmE involves *slyA.* (A) The pPROBE-AT plasmid harboring the *slyA*-GFP promoter region was measured by flow cytometry. *slyA* promoter activity in the wild type, Δ*ecpC*, Δ*vfmE*, Δ*vfmE* Δ*ecpC*, Δ*vfmP*, and Δ*vfmP* Δ*ecpC* was measured. Mean fluorescence intensity (MFI), and average GFP fluorescence intensity of total bacterial cells were examined. The experiment was performed with three replicates. Labels above the bars indicate statistically significant differences between treatments for 24 h in MM medium (*P* < 0.05) by one-way ANOVA. (B) The total Pectate Lyase activity was measured by spectrophotometry. The total Pel activity in wild-type, Δ*ecpC*, Δ*vfmE*, Δ*vfmE* Δ*ecpC*, Δ*slyA*, Δ*slyA* Δ*ecpC*, Δ*vfmE* Δ*slyA*, and Δ*vfmE* Δ*slyA* Δ*ecpC* was measured. The OD_230_ value of Pel activity was normalized by OD_600_ value of the cell culture. Values are representative of two experiments, and three replicates were used for each experiment. The lowercase letters represent different treatment groups with significant statistical differences, whereas treatments with no significant differences were assigned the same letters for 16 h in LB medium (*P* < 0.05) by one-way ANOVA.

### VfmE controls pectate lyase activity through the transcriptional regulator SlyA.

SlyA, a regulator of the SlyA/MarR family, was reported to be an important regulator of virulence genes, and it upregulates pectate lyase production in *D. dadantii* (14, 15). In Dickeya zeae, VfmE positively regulates *slyA* (16). In order to elucidate whether *slyA* is involved in the pathway of *pelD* regulation by VfmE, we checked the promoter activity of *slyA* in Δ*vfmE* mutants. We found that Δ*vfmE* and Δ*vfmP* single deletion mutants showed a reduced *slyA* promoter activity (Fig. 3). The *ecpC* single mutant also showed reduced *slyA* activity. Surprisingly, double deletion of *vfmE*/*vfmP* and *ecpC* did not show a further reduction in *slyA* promoter activity but, rather, restored the *slyA* expression back to the WT level (Fig. 3A). This suggests that even though VfmE/VfmP positively regulates *slyA* transcriptionally in the wild-type background, such positive regulation was reversed to become a negative regulation when *ecpC* was mutated, which is analogous to the *pelD* expression observed in these strains (Fig. 1A).

Since the expression of *pelD* and *slyA* both showed inverse phenotypes in Δ*vfmE* and Δ*vfmE* Δ*ecpC* mutant backgrounds, we investigated whether the restored *slyA* transcription in Δ*vfmE* Δ*ecpC* was the reason for the restored extracellular Pel activity. Chromosomal deletion mutants of *slyA* were constructed in WT, Δ*ecpC*, Δ*vfmE*, and Δ*vfmE* Δ*ecpC* backgrounds, and the total extracellular *pel* activity was measured. Compared to the WT, reduced *pel* activities were detected in Δ*ecpC*, Δ*vfmE*, Δ*slyA*, Δ*slyA* Δ*ecpC*, and Δ*vfmE* Δ*slyA* mutants. The reduced *pel* activity in single deletion mutants of *vfmE* and *ecpC* was restored to the WT level in the Δ*vfmE* Δ*ecpC* double mutant. However, such restoration was abolished by an additional mutation of *slyA* in the triple deletion mutant Δ*vfmE* Δ*slyA* Δ*ecpC*, which showed further reduction in Pel activity compared to the Δ*ecpC*, Δ*vfmE*, Δ*slyA*, Δ*slyA* Δ*ecpC*, and Δ*vfmE* Δ*slyA* mutants (Fig. 3B). These data suggest that the upregulation of *pelD* promoter activity or total Pel activity in the Δ*vfmE* Δ*ecpC* double mutant could be attributed to the increased transcription of *slyA.*

### VfmE is regulated by the cellular c-di-GMP regulatory system.

**FIG 4:**
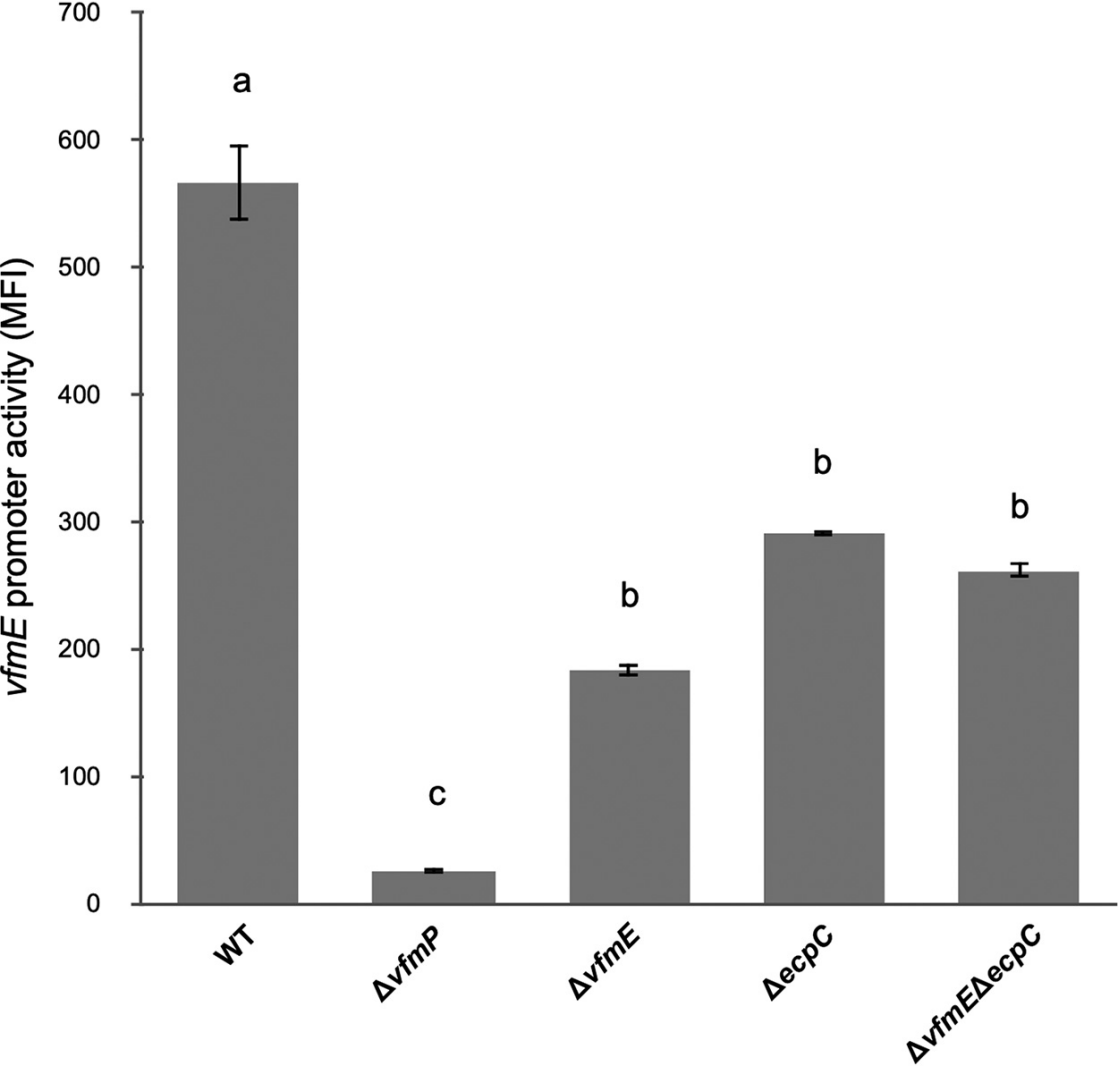
EcpC regulates *vfmE* at the transcriptional level. The promoter activity of pPROBE-AT plasmid harboring *vfmE-*GFP promoter region was measured by flow cytometry. The *vfmE* promoter activities were measured in the wild type, Δ*vfmP*, Δ*vfmE*, Δ*ecpC*, and Δ*vfmE* Δ*ecpC* mutants. Mean fluorescence intensity (MFI) and average GFP fluorescence intensity of total bacterial cells were examined. The experiment was performed with three replicates. The lowercase letters represent different treatment groups with significant statistical differences, whereas treatments with no significant differences were assigned the same letters for 24 h in MM medium (*P* < 0.05) by one-way ANOVA.

As EcpC is a phosphodiesterase that degrades intracellular c-di-GMP, the intracellular c-di-GMP level in Δ*ecpC* is 2 times higher than that in the WT as previously determined (2). Under such conditions, the *vfmE* promoter activity was significantly reduced in the Δ*ecpC* mutant compared to the WT. The promoter activity of *vfmE* was validated in Δ*vfmP* and in Δ*vfmE*; in both strains, the *vfmE* promoter activity showed a drastic reduction compared to the WT. This is expected because VfmP is the synthase of the quorum-sensing signaling molecule, and VfmE, in the presence of the quorum-sensing signaling molecules, activates its own transcription (13). The Δ*vfmE* Δ*ecpC* mutant showed a similar level of *vfmE* transcription as the Δ*ecpC* mutant (Fig. 4). These data indicate that the transcription of *vfmE* was regulated by EcpC and VfmE via the same pathway. We observed a lower *vfmE* transcription in the Δ*vfmP* mutant than in the Δ*vfmE* mutant because the Δ*vfmP* mutant shuts off the entire Vfm quorum-sensing system and production of quorum-sensing molecules. However, in the Δ*vfmE* mutant, a smaller amount of VfmH is produced (13), which can transcribe the *vfmE* promoter at a lower level.

### Multiple sequence alignment revealed a potential c-di-GMP binding RxxxR motif in VfmE.

**FIG 5:**
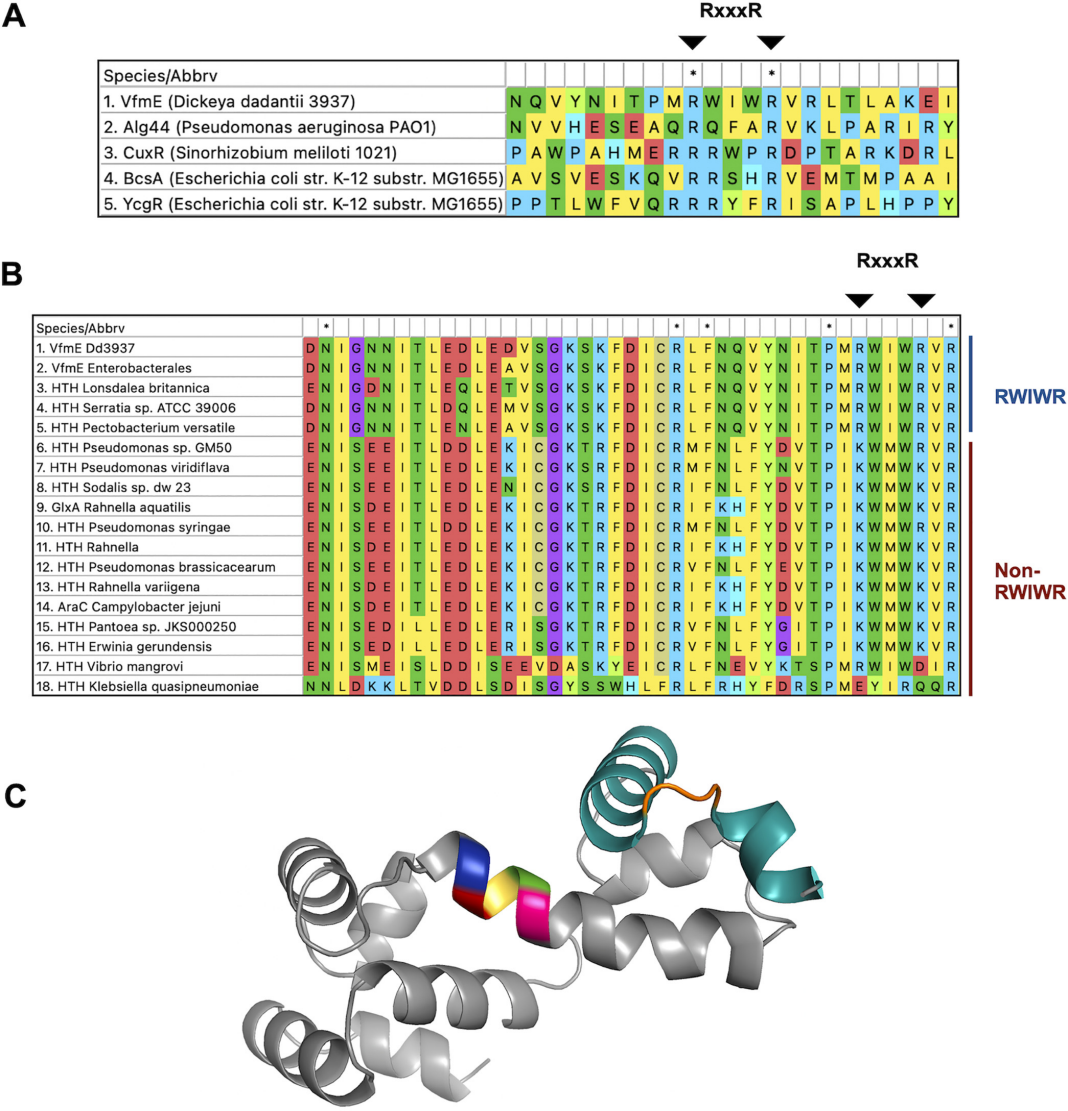
The conserved RxxxR c-di-GMP binding motif of VfmE and other c-di-GMP binding proteins with RxxxR motifs. (A) The multiple sequence alignment of VfmE (GenBank accession version no. WP_013320004.1) with other known RxxxR motifs containing c-di-GMP binding proteins Alg44 (WP_134306546.1), CuxR (WP_010975317.1), DgrA (WP_003230551.1), BcsA (NP_417990.4), and YcgR (NP_415712.1) showing the aligned RxxxR motif. Asterisks indicate the conserved amino acid residues. (B) The multiple sequence alignment of VfmE and other AraC family proteins across various bacteria. The ones containing the RWIWR motif are labeled separately from the ones without the RWIWR motif. Asterisks indicate the conserved amino acid residues. (C) A homology modeling of VfmE was done (36). The predicted c-di-GMP binding RWIWR motif is colored as follows: blue, Arg89; red, Trp90; yellow, Ile91; green, Trp92; magenta, Arg93. The C-terminal helix-turn-helix DNA binding domain is colored teal, orange, and teal, respectively.

We hypothesized that VfmE is a c-di-GMP binding protein, the function of which is highly dependent on the intracellular c-di-GMP concentrations. Also, as the *vfmE* promoter is regulated by VfmE itself, the repression of *vfmE* transcription by high c-di-GMP (Δ*ecpC*) can be potentially through inactivation of the activator VfmE. Many proteins, such as YcgR in Escherichia coli and CuxR in Sinorhizobium meliloti, bind to c-di-GMP through the RxxxR motif (5, 6, 17–19). To determine if such an RxxxR signature motif also exists in VfmE, multiple sequence alignment of VfmE (GenBank version no. WP_013320004.1) with other known RxxxR motifs containing c-di-GMP binding proteins Alg44 (WP_134306546.1), CuxR (WP_010975317.1), DgrA (WP_003230551.1), BcsA (NP_417990.4), and YcgR (NP_415712.1) was performed. Although an RxxxR sequence was identified in VfmE, the amino acid sequences flanking the motif are highly diversified (Fig. 5A). We used the AraC domain of VfmE to search against the NCBI protein database and have identified multiple homologues in various species, with only a minority of them containing the RWIWR motif (Fig. 5B). This suggests that the RWIWR motif may have evolved in the AraC family regulators to adapt these proteins to bind c-di-GMP. Using the protein structure predicting tool Phyre2, the RWIWR motif was identified at the N-terminal end of the protein. A helix-turn-helix domain, with a putative DNA binding function, was identified at the C-terminal end (Fig. 5C).

### c-di-GMP binds to WT VfmE but not VfmE^R93D^.

**FIG 6:**
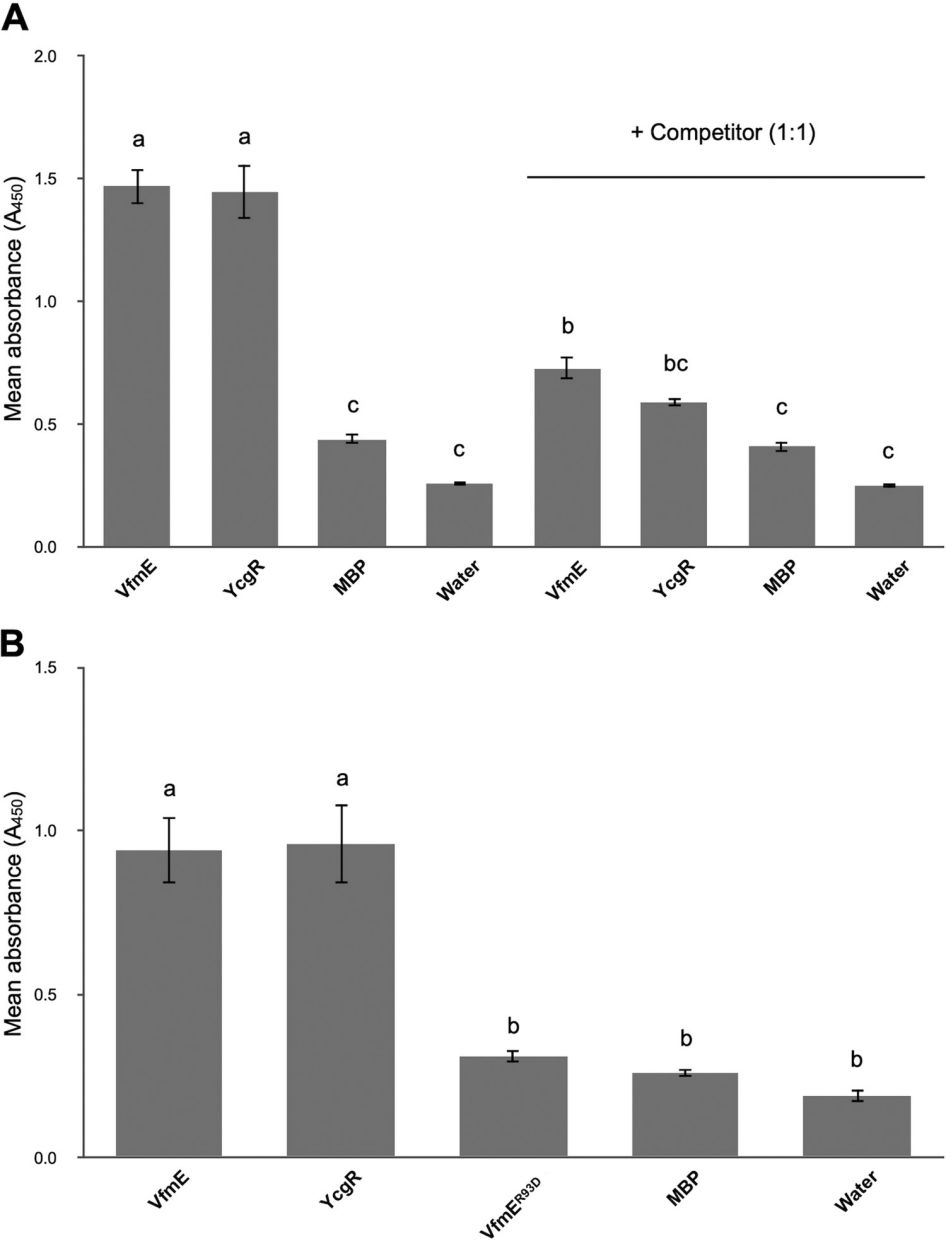
VfmE is a c-di-GMP binding protein. The c-di-GMP binding ability of VfmE was determined by ELISA. (A) Absorbance at 450 nm observed in VfmE, using YcgR, MBP, and sterile water as controls. A similar experiment was done by adding nonlabeled c-di-GMP as a specific competitor. (B) Absorbance at 450 nm observed in VfmE and VfmE^R93D^, keeping YcgR, MBP, and sterile water as controls. Values are representative of two independent experiments, and three replicates were used for each experiment. The lowercase letters represent different treatment groups with significant statistical differences, whereas treatments with no significant differences were assigned the same letters (*P* < 0.05) by one-way ANOVA.

To determine if VfmE indeed contains c-di-GMP binding ability, we performed a modified enzyme-linked immunosorbent assay (ELISA) (20). In brief, wells of an ELISA plate were coated first with VfmE protein, followed by biotinylated c-di-GMP. The binding of the biotin-labeled c-di-GMP was detected by horseradish peroxidase (HRP)-conjugated streptavidin with TMB (3,3′,5,5′-tetramethylbenzidine) coloring substrate. HRP produced a measurable color change of TMB, and the color intensity was detected by a plate reader at 450 nm. For this experiment, a known c-di-GMP effector, YcgR from *D. dadantii*, was used as a positive control (2), and maltose binding protein (MBP) was used as a negative control (21). Wells containing only sterile water were used for the reference of background binding. We observed a high level of 450 nm absorbance in VfmE- and YcgR-treated wells and a low level of 450 nm absorbance in the MBP and water controls (Fig. 6A). To confirm the specificity of the c-di-GMP binding, we performed a competition assay where we added an equal concentration of unlabeled c-di-GMP to the wells. We observed a reduced absorbance in both VfmE- and YcgR-treated wells, whereas no reduction was observed in MBP- and water-treated wells. This result reveals that VfmE binds c-di-GMP with specificity comparable to that of a known c-di-GMP effector, YcgR.

To determine if the identified RWIWR motif is essential for the c-di-GMP binding of VfmE, we constructed a single amino acid substitution at the 93rd position of VfmE, which changed the RWIWR motif to RWIWD. The purified VfmE^R93D^ protein was tested in the c-di-GMP binding assay. Compared to a high 450 nm absorbance observed in YcgR- and VfmE-treated wells, a reduced 450 nm absorbance was observed in VfmE^R93D^-treated wells at a similar level of the MBP and water controls (Fig. 6B). This confirms that the Arg^93^ motif in VfmE is indeed essential for c-di-GMP binding.

### VfmE^R93D^ recovers the Pel production regardless of high-c-di-GMP conditions.

**FIG 7:**
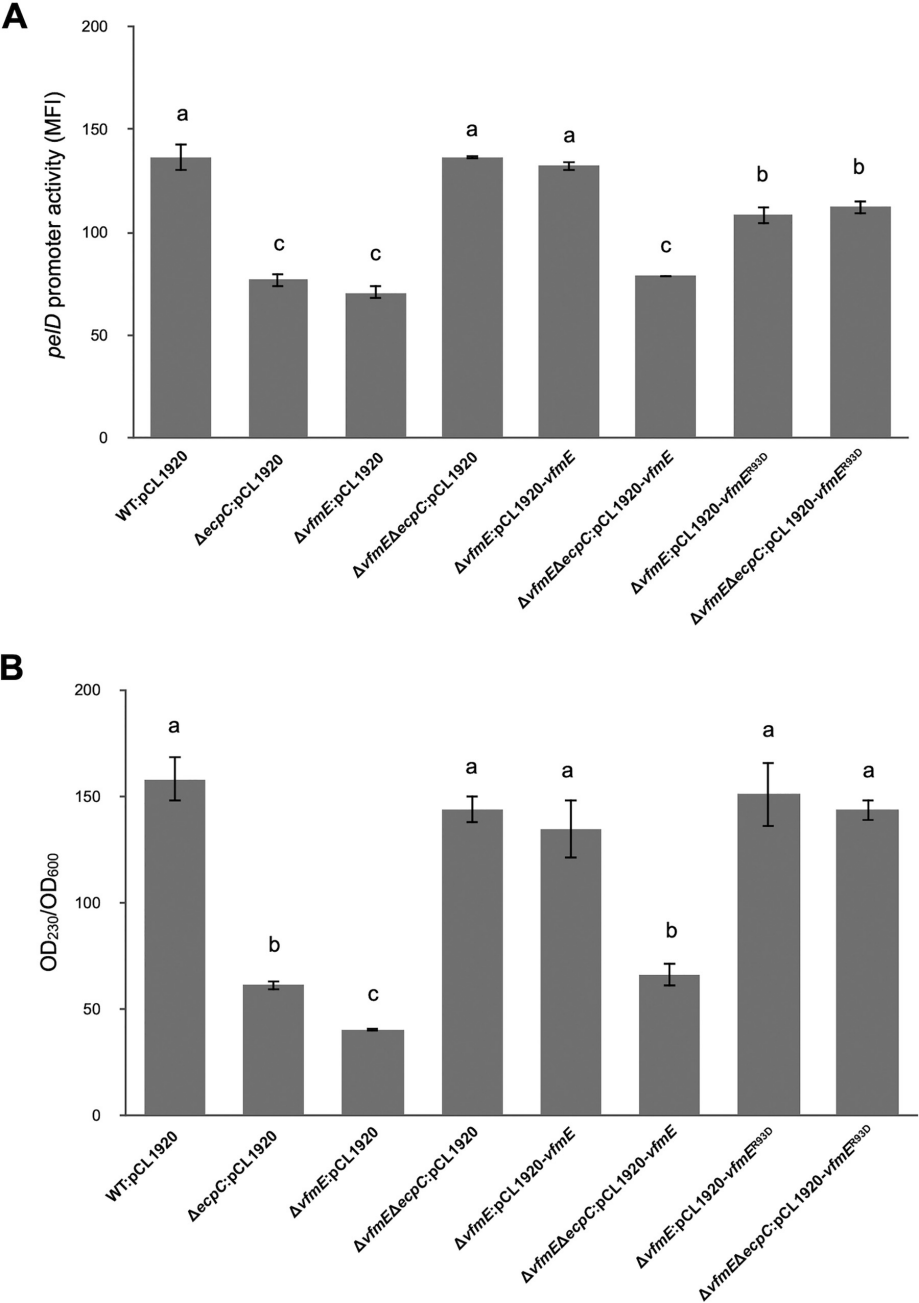
VfmE protein with an R93D substitution differentially affects pectate lyase activity under the Δ*ecpC* mutant. (A) The promoter activity of the pPROBE-AT plasmid harboring the *pelD*-GFP promoter region was measured by flow cytometry. *pelD* promoter activity in the wild type, Δ*ecpC*, Δ*vfmE*, and Δ*vfmE* Δ*ecpC* was measured. Complementation of *pelD* promoter activity in the wild type, Δ*ecpC*, Δ*vfmE*, and Δ*vfmE* Δ*ecpC* harboring low-copy-number empty pCL1920 plasmid (controls) and Δ*vfmE* and Δ*vfmE* Δ*ecpC* strains harboring pCL1920:*vfmE* and pCL1920:*vfmE*^R93D^. Values are representative of three experiments, and three replicates were used for each experiment. Mean fluorescence intensity (MFI) and average GFP fluorescence intensity of total bacterial cells were examined. The lowercase letters represent different treatment groups with significant statistical differences, whereas treatments with no significant differences were assigned the same letters for 24 h in MM medium (*P* < 0.05) by one-way ANOVA. (B) The total pectate lyase activity was measured by spectrophotometry. Complementation of *pelD* promoter activity in the wild type, Δ*ecpC*, Δ*vfmE*, and Δ*vfmE* Δ*ecpC* harboring low-copy-number empty pCL1920 plasmid (controls) and Δ*vfmE*, and Δ*vfmE* Δ*ecpC* strains harboring pCL1920:*vfmE*, and pCL1920:*vfmE*^R93D^. The total Pel activity in the wild type, Δ*ecpC*, Δ*vfmE*, and Δ*vfmE* Δ*ecpC* strains was measured. The OD_230_ value of Pel activity was normalized by the OD_600_ value of the cell culture. Values are representative of three experiments, and three replicates were used for each experiment. The lowercase letters represent different treatment groups with significant statistical differences, whereas treatments with no significant differences were assigned the same letters for 16 h in LB medium (*P* < 0.05) by one-way ANOVA.

To investigate the regulatory mechanism of VfmE on *pel* expression under high-c-di-GMP conditions, we tested the *pelD* promoter activities of the WT and the Δ*ecpC*, Δ*vfmE*, and Δ*vfmE* Δ*ecpC* mutants and complemented them with *vfmE*^WT^ or *vfmE*^R93D^ cloned into plasmid pCL1920 under the native promoter of *vfmE*. Consistent with previous results, Δ*ecpC* and Δ*vfmE* showed reduced *pelD* expression compared to the WT, and Δ*vfmE* Δ*ecpC* showed expression on par with the WT. The plasmid containing the *vfmE*^WT^ gene complemented the *pelD* expression in Δ*vfmE* to the WT level and Δ*vfmE* Δ*ecpC* to the Δ*ecpC* level. However, *pelD* expression was on par with the WT level when the plasmid containing *vfmE*^R93D^ was transformed into Δ*vfmE* Δ*ecpC*, suggesting VfmE^R93D^ recovered the *pelD* expression regardless of the high c-di-GMP conditions in Δ*ecpC* (Fig. 7A).

We further confirmed the finding by performing the extracellular Pel activity. The total extracellular Pel activity was measured by spectrophotometry using 16-h cultures (7). We observed reduced Pel activities for Δ*ecpC* and Δ*vfmE* mutants. The Pel activity in Δ*vfmE* Δ*ecpC* was higher than that in either the Δ*ecpC* or Δ*vfmE* mutant and on par with the WT level. The Pel activity was complemented in Δ*vfmE* (to WT level) and Δ*vfmE* Δ*ecpC* (to Δ*ecpC* level) with the plasmid containing the *vfmE*^WT^ gene. Similar to the *pelD* expression result, *vfmE*^R93D^ recovered the Pel activity in Δ*vfmE* and Δ*vfmE* Δ*ecpC* to the WT level regardless of high-c-di-GMP conditions (Fig. 7B). The above-described results demonstrated that VfmE is a positive regulator of Pel activity in *D. dadantii*, and the c-di-GMP binding motif mediates binding to c-di-GMP to convert VfmE to a repressor at higher c-di-GMP concentrations.

Since in *trans* expression of *vfmE*^R93D^ recovered the Pel activity to WT levels regardless of the high c-di-GMP conditions (Δ*ecpC*), we checked the effect on virulence by infection assay in potato. Similar to the result in Pel activity, *vfmE*^R93D^ recovered the virulence in Δ*vfmE* Δ*ecpC* to the WT level regardless of high c-di-GMP background (Fig. 2). This further confirms that the Arg^93^ in VfmE is essential to repress virulence under high-c-di-GMP conditions. To check the survival of the mutants in the host, the fluid from the necrotic tissue was collected, and the CFU of each of the strains was measured. We observed that Δ*ecpC* and Δ*vfmE* mutants had 10-fold lower CFU count in the host. Similar to the finding of Pel activity, Δ*vfmE* Δ*ecpC* had a count CFU similar to that of the WT. Complementation with *vfmE* in the Δ*vfmE* Δ*ecpC* mutant resulted in a CFU count similar to that of Δ*ecpC*, while complementation with *vfmE*^R93D^ in this strain resulted in a CFU count similar to that of the WT (see Fig. S1 in the supplemental material). These results show that c-di-GMP binding is important for VfmE function *in vivo*. VfmE takes part in virulence and survival in the host, and the c-di-GMP binding motif in VfmE is a key determinant of VfmE function in response to c-di-GMP.

## DISCUSSION

**FIG 8:**
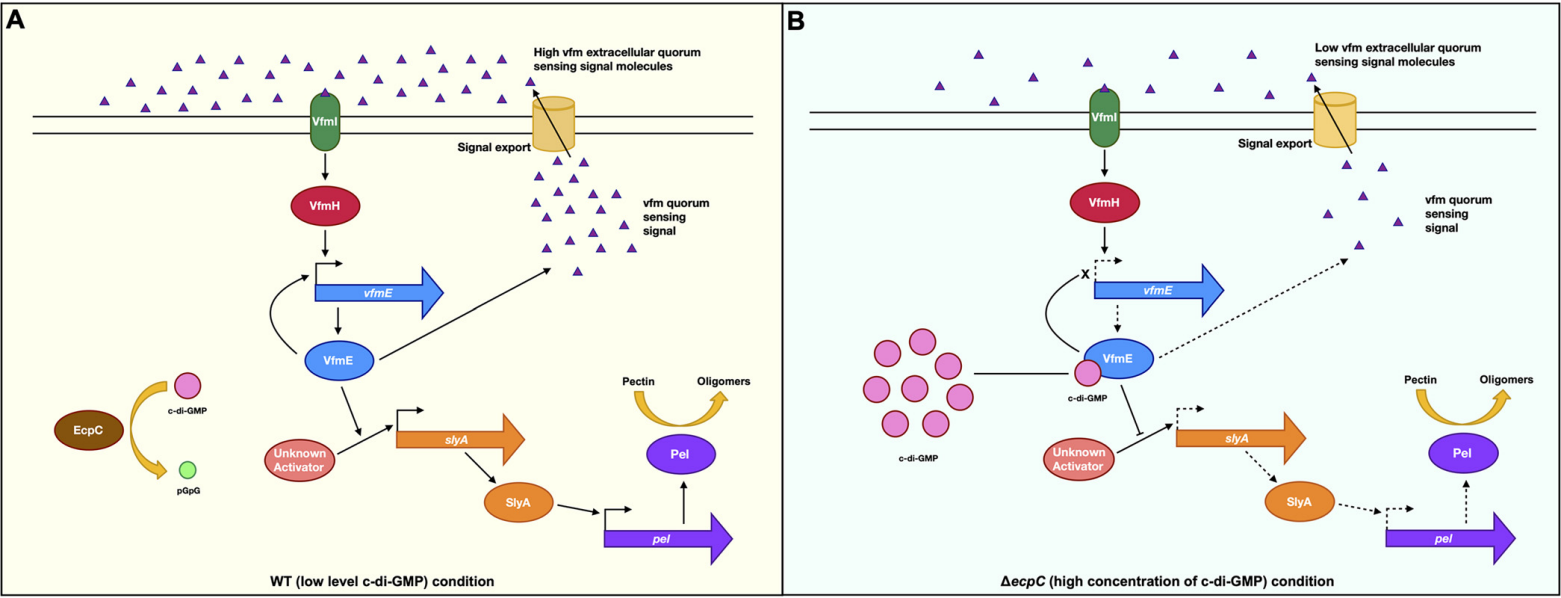
Regulatory mechanism of VfmE. The regulatory mechanisms of VfmE on Pel production under low and high c-di-GMP conditions. (A) VfmE upregulates *pel* production through *slyA* transcription, resulting in a high Pel activity when the c-di-GMP level is the WT (low) level. Under this condition, VfmE also upregulates its own transcription, resulting in high *vfm* quorum-sensing molecule production, which corresponds to high *vfmE* transcription. (B) Under a high c-di-GMP condition (i.e., Δ*ecpC*), c-di-GMP-bound VfmE represses the unidentified activator of *slyA*, resulting in low *slyA* transcription and Pel activity. High c-di-GMP caused by deletion of *ecpC* also reduces *vfmE* transcription, resulting in low *vfm* quorum-sensing molecule production, which corresponds to low *vfmE* transcription. Solid arrows indicate higher production of SlyA, Pel, *vfmE*, and *vfm* quorum-sensing molecules; dotted arrows indicate lower production of SlyA, Pel, *vfmE*, and *vfm* quorum-sensing molecules.

Although in a previous study, a single knockout of *vfmE* decreased the expression of *pel* genes (13), transposon insertion into *vfmE* in the Δ*ecpC* background unexpectedly recovered the reduced *pelD* promoter activity to the WT level (Fig. 1A and B). Previously, a MarR family transcriptional regulator, SlyA, was reported to positively regulate Pel production and virulence in *D. dadantii* (14, 15) and was positively regulated by VfmE in *D. zeae* (16). Consistent with the previous finding, *vfmE* positively regulates *slyA* transcription. However, similar to the finding of *pelD* expression in the Δ*vfmE* Δ*ecpC* mutant, the *slyA* expression was also recovered to the WT level in Δ*vfmE* Δ*ecpC* compared to Δ*vfmE* or Δ*ecpC* single mutants (Fig. 3A). We suggest that the recovery of Pel activity in the Δ*vfmE* Δ*ecpC* double mutant was due to upregulated *slyA* transcription. The Δ*vfmE*, Δ*slyA*, and Δ*vfmE* Δ*slyA* mutants showed a similar level of Pel activities, indicating that VfmE and SlyA are in the same regulatory pathway. Further, deletion of *slyA* in the Δ*ecpC* background did not recover the Pel activities as seen in the Δ*vfmE* Δ*ecpC* mutant (Fig. 3B), and deletion of *slyA* in the Δ*vfmE* Δ*ecpC* mutant suppressed the recovery phenotype of the Δ*vfmE* Δ*ecpC* mutant in the Pel activity (Fig. 3B). These results suggest that VfmE positively regulates Pel activity in low-c-di-GMP conditions through activation of SlyA, and this function is inhibited under high-c-di-GMP conditions (i.e., Δ*ecpC*) (Fig. 8). We speculate the presence of an additional unidentified transcriptional activator of *slyA*, which is repressed by VfmE under high-c-di-GMP conditions but can activate *slyA* when *vfmE* is deleted under the Δ*ecpC* mutant.

A reduced pectate lyase activity was observed in the triple mutant, Δ*vfmE* Δ*slyA* Δ*ecpC*, compared to the double mutant Δ*vfmE* Δ*slyA*. It is possible that the decreased pectate lyase synthesis is caused by high c-di-GMP levels due to the *ecpC* mutation in the triple mutant. Furthermore, the differences in Pel activities in Δ*vfmE* and Δ*slyA* mutants suggest that VfmE regulates pel transcription not only via *slyA* but also via an additional regulator, although these differences are not statistically significant. SlyA plays an important role in this regulatory pathway because losing *slyA* suppresses Pel activity, and it cannot be recovered in *vfmE* and *ecpC* double deletion. We speculate that *slyA* expression is increased if *vfmE* is deleted under high-c-di-GMP conditions (Δ*ecpC*); however, further investigation is required to resolve this linkage. The smaller maceration areas in the *vfmE* and *ecpC* mutants were observed because of lower pectate lyase production, although we cannot rule out the possibility that the slower growth of the *vfmE* mutant in the potato may also be a partial cause.

As VfmE is an activator of its own transcription (13) and Δ*vfmE* and Δ*vfmE* Δ*ecpC* mutants showed similar *vfmE* transcription, we hypothesize that under high-c-di-GMP conditions, c-di-GMP binding inhibits VfmE activity. Previous research indicated that AraC family transcriptional regulators can bind c-di-GMP via the RxxxR motif (19), and a conserved RWIWR motif was also found in VfmE by multiple sequence alignment (Fig. 5A). The c-di-GMP binding ELISA shows that VfmE binds c-di-GMP (Fig. 6A). Further, swapping the Arg^93^ residue to an aspartate residue in the RWIWR^93^ motif (RWIWD^93^) abolishes the binding of VfmE to c-di-GMP (Fig. 6B). Our *in vitro* ELISA binding experiments and *in vivo* phenotypic assay are consistent with VfmE binding c-di-GMP via the RWIWR motif, similar to the PilZ-like mechanism.

VfmE was previously reported to regulate several DGCs and PDEs in *D. zeae* (16). Our results show that VfmE positively regulates a DGC, *gcpA* (Fig. S2A), and a PDE, *egcpB*, at the transcriptional level (Fig. S2B) but does not directly regulate PDE *ecpC* transcription (Fig. S2C). Although VfmE was found to control some c-di-GMP regulatory genes, the intracellular c-di-GMP level remains unaffected in the Δ*vfmE* mutant (Fig. S2D). This implies that VfmE does not have a dominant role in regulating overall cellular c-di-GMP levels under the conditions tested.

High c-di-GMP levels have been previously reported to antagonize quorum sensing in Burkholderia cenocepacia H111 and Pseudomonas spp. (22–24). In this study, we observed that the high c-di-GMP concentration caused by deletion of *ecpC* represses *vfmE* transcription (Fig. 4). Since VfmE is a self-activator, the above-described observation indicates that c-di-GMP inhibits the transcription of *vfmE* via VfmE. Reduction in *vfmE* transcription indicates reduction of the transcription levels of the *vfm* gene cluster (13). This trend is consistent with our observations of c-di-GMP and VfmE on *pel* transcription (Fig. 8). Since the transcription of *vfmE* is dependent on the *vfm* quorum sensing, low transcription of *vfmE* is an indication of reduced *vfm* quorum sensing under the *ecpC* deletion mutant (high c-di-GMP condition). This hypothesis is verified by the mutant VfmE^R93D^, which is able to recover the Δ*vfmE* phenotypes in normal c-di-GMP levels and in a high-c-di-GMP environment of Δ*ecpC* background (Fig. 7A to B). VfmE^R93D^ displays the same molecular function as VfmE^WT^ but is insensitive to c-di-GMP, which does not allow the cells to appropriately respond to the high-c-di-GMP environment. This also suggests that the C-terminal helix-turn-helix domain of VfmE can work independently to activate its regulons, while the role of the c-di-GMP binding motif RWIWR is for suppression of the activity.

We propose that Vfm signal induces the transcription of *vfmE* to regulate Pel production, and high c-di-GMP levels suppress VfmE activity through the binding of RWIWR. Thus, VfmE is one of the activators of Pel but acts as a repressor when the c-di-GMP concentration is high. c-di-GMP is a ubiquitous bacterial second messenger that regulates multiple cellular functions, such as virulence. Given that VfmE is a key member in Vfm quorum sensing, this study has provided a strong link between quorum sensing and c-di-GMP signaling to mediate various responses to environmental changes.

## MATERIALS AND METHODS

### Bacterial strains, plasmids, primers, and media.

The bacterial strains and plasmids used in this study are listed in Table S1. *D. dadantii* 3937 and mutant strains were stored at −80°C in 40% glycerol. *D. dadantii* strains were grown in Luria-Bertani (LB) medium (1% tryptone, 0.5% yeast extract, and 1% NaCl), mannitol-glutamic acid (MG) medium (1% mannitol, 0.2% glutamic acid, 0.05% potassium phosphate monobasic, 0.02% NaCl, and 0.02% MgSO_4_), or low-nutrient minimal medium (MM) at 28°C (25, 26). Escherichia coli strains were grown in LB at 37°C. Antibiotics were added to the media at the following concentrations: 100 μg mL^−1^ ampicillin, 50 μg mL^−1^ kanamycin, and streptomycin. The *D. dadantii* 3937 genome sequence can be retrieved from a systematic annotation package for community analysis of genomes (ASAP) (https://asap.ahabs.wisc.edu/asap/home.php). The primers used for PCR in this report are listed in Table S2.

### Mutant construction and complementation.

The *vfmE* and *vfmP* genes were deleted from the genome by marker exchange mutagenesis (27). Briefly, two fragments flanking each target gene were amplified by PCR with specific primers (Table S2). The kanamycin (Km) cassette was amplified from pKD4 (28) and was cloned between two flanking regions using three-way crossover PCR. The PCR construct was inserted into the suicide plasmid pWM91, and the resulting plasmid was transformed into *D. dadantii* 3937 by conjugation using E. coli strain S17-1 λ-pir. To select strains with chromosomal deletions, recombinants grown on a kanamycin medium were plated on a 5% sucrose plate. Cells that were resistant to sucrose due to SacB-mediated toxicity were then plated on an ampicillin plate, and the ampicillin-sensitive cells were confirmed by PCR using outside primers. Finally, the DNA fragment, which contains two flanking regions and a kanamycin cassette, was sequencing confirmed. Markerless mutants were constructed by excision of the Km cassette from the marker exchange mutants using pFLP2 plasmid encoding the FLP (flippase) recombinase enzyme in E. coli S17-1 λ-*pir*. After conjugation, the strains sensitive to kanamycin and resistant to sucrose were selected and analyzed by sequencing using outside primers. For constructing the *slyA* mutants, the pWM91:*slyA* plasmid from previous research was used (29). To generate complemented strains, the promoter and open reading frame region of target genes were amplified and cloned into low-copy-number plasmid pCL1920 (Table S1). The resulting plasmids were then confirmed by PCR and electroporated into mutant cells.

### Green fluorescent protein (GFP) reporter plasmid construction and flow cytometry assay.

To generate the reporter plasmids, pAT-*vfmE*, the promoter region of *vfmE* was PCR amplified and cloned into the promoter probe vector pPROBE-AT, which contains the ribosomal binding site upstream of the *gfp* gene (30, 31). The reporter plasmids pAT-*pelD*, pAT-*slyA*, pAT-*gcpA*, pAT-*egcpB*, and pAT-*ecpC* were constructed previously following the same procedure (8, 29, 32, 33). Promoter activity was monitored by measuring GFP intensity through flow cytometry (BD Biosciences, San Jose, CA) as previously described (33). Briefly, bacterial cells with reporter plasmid were grown in LB medium overnight and inoculated 1:100 into MM. Samples were collected at 24 h, and promoter activity was analyzed by detecting GFP intensity using flow cytometry. The *pelD* promoter activity was measured with bacterial cultures grown for 24 h in MM supplemented with 0.1% polygalacturonic acid (PGA).

### Pectate lyase activity assay.

Extracellular Pel activity was measured by spectrometry as previously described (34). Briefly, bacterial cells were grown in LB medium supplemented with 0.1% polygalacturonic acid at 28°C for 16 h. For extracellular Pel activity, 1-mL bacterial cultures were centrifuged at 15,000 rpm for 2 min, the supernatant was then collected, and 10 μL of the supernatant was added to 990 μL of the reaction buffer (0.05% PGA, 0.1 M Tris-HCl [pH 8.5], and 0.1 mM CaCl_2_, prewarmed to 30°C). Pel activity was monitored at an absorbance of 230 nm (A_230_) for 3 min and calculated based on one unit of Pel activity equaling an increase of 1 × 10^−3^ optical density at 230 nm (OD_230_) in 1 min. The cell density OD_600_ value of the liquid cell cultures of each sample was measured. The final value of OD_230_ was normalized by the OD_600_ value of each of the sample cultures (OD_230_/OD_600_).

### Virulence assay.

The local maceration assay was performed using russet potato (Solanum tuberosum) as described (8). For each potato, 50 μL of a bacterial suspension at 10^6^ CFU mL^−1^ was syringe infiltrated in the middle of each sliced half of 1 cm thickness. Phosphate buffer (50 mM, pH 7.4) was used to suspend the bacterial cells. A potato in three replicates was used for each bacterial strain. Inoculated potato slices were kept in a growth chamber at 28°C with 100% relative humidity. To evaluate the necrosis of potato tissue, the soft necrotic tissue was scooped out, and the weight was measured for each sample. The fluid from the necrotic tissue was collected (1 mL), serial dilution was done, and the CFU count of each of the strains was obtained to determine the survival ability of the strains in the host system.

### Construction of point mutation.

The full length of *vfmE* along with its natural promoter was cloned into the low-copy-number plasmid pCL1920 by primers vfmE-F-HindIII and vfmE-R-XbaI (Table S2). To construct the point-specific mutation in the RxxxR motif of VfmE protein, single nucleotide substitution was performed by amplifying two fragments containing the desired mutation on both strands and then joining them into one by fusion PCR. Briefly, a primer set, vfmE-R93D-1 and vfmE-R93D-2 (Table S2), was used to generate *vfmE*^R93D^, which changed the RxxxR motif to RxxxD. The substitution was confirmed by DNA sequencing. The pCL1920:*vfmE*^R93D^ plasmid construct was used to check the complementation of the *pelD* promoter activity and total Pel activity in Δ*vfmE* and Δ*vfmE* Δ*ecpC* mutant backgrounds. The pMAL-c6T:*vfmE*^R93D^ plasmid was used to overexpress the mutated protein for the c-di-GMP binding ELISA.

### Protein expression and purification.

The full lengths of *vfmE* and *vfmE*^R93D^ were cloned into the expression vector pMAL-c6T (New England Biolabs) by primers vfmE-F-BamHI and vfmE-R-HindIII (Table S2) under *tac* promoter. The sequences of the constructs were verified by DNA sequencing. The constructs carrying *vfmE* and *vfmE*^R93D^ were transformed into NEBExpress E. coli competent cells for protein expression and purification. NEBExpress E. coli cells containing empty pMAL-c6T, which expresses only 6×-His-MBP, was used as the control. Briefly, expression of fusion proteins was induced by the addition of isopropyl-thio-galactopyranoside (IPTG) at a final concentration of 0.1 mM, and the bacterial cultures were then incubated at 30°C for 4 h. Then bacterial cells were collected by centrifugation, followed by suspension in MBP column binding buffer and sonication. The crude cell extracts were centrifuged at 15,000 rpm for 15 min to remove cell debris. The supernatant containing the soluble proteins was analyzed by sodium dodecyl sulfate polyacrylamide gel electrophoresis and Western blotting using anti-MBP monoclonal primary antibody (New England Biolabs) and anti-mouse IgG-HRP conjugate secondary antibody (Southern Biotech). The presence of full-length 6×-His-MBP-VfmE and 6×-His-MBP-VfmE^R93D^ in the cell lysate was further confirmed by mass spectrometry (University of Wisconsin-Madison). A His-tagged variant of YcgR from *D. dadantii* was purified by the method used previously (2). The cell lysate containing the recombinant protein was analyzed by sodium dodecyl sulfate polyacrylamide gel electrophoresis and Western blotting using HRP-conjugated anti-His monoclonal antibody (Invitrogen). The supernatant of the cell lysates containing the soluble proteins was used for the c-di-GMP binding assay.

### c-di-GMP binding assay.

The assay was modified from the c-di-AMP binding protein detection assay (20). The protein concentrations in the cell lysates containing 6×-His-MBP-VfmE, 6×-His-MBP-VfmE^R93D^, 6×-His-MBP, and 6×-His-YcgR were measured by standard Bradford assay and then diluted to 50 μg/mL in coating buffer containing 3.03 g Na_2_CO_3_, 6.0 g NaHCO_3_ in 1 L water, pH 9.6. Immulon 96-well plates (Thermo Fisher Scientific) were coated with the diluted proteins (100 μL/well) overnight (16 h) at 4°C. The wells were blocked with 100 μL 1% bovine serum albumin in phosphate-buffered saline (pH 7.4) for 1 h at room temperature. Then 100 μL of 25 nM biotinylated c-di-GMP (Biolog) was added to each well and incubated at room temperature for 2 h. Next, 100 μL of 1:15,000 HRP-conjugated streptavidin (Thermo Scientific) was added to the wells and incubated at room temperature for 1 h. The wells were washed three times with 200 μL phosphate-buffered saline (pH 7.4) containing Tween 20 (PBST) in between every step. Then, 100 μL of Pierce TMB substrate (Thermo Fisher Scientific) was added to each well and incubated for 30 min at room temperature. The colorimetric reaction was stopped by adding 100 μL 2 M H_2_SO_4_. The absorbance was measured at 450 nm using a plate reader. For the competition assay, 10 μL of 250 nM unlabeled c-di-GMP (Biolog) was added to each well. For control of the competition assay, 10 μL sterile water was added to each of the samples.

### Measurement of intracellular c-di-GMP levels.

Intracellular c-di-GMP concentrations were determined by using high-performance liquid chromatography coupled with tandem mass spectrometry (HPLC-MS/MS), which has been described previously (35). Overnight bacterial cultures were inoculated 1:100 into 30 mL LB medium in a flask. When the OD_600_ of the bacterial culture reached about 0.8, corresponding to mid- to late-exponential growth, all cells were centrifuged in 50-mL polystyrene centrifuge tubes for 30 min at 4,000 rpm. The supernatant was then discarded, and the pellet was resuspended in 1.5 mL extraction buffer (40% acetonitrile, 40% methanol in 0.1 N formic acid). To release intracellular c-di-GMP, cells resuspended in extraction buffer were left at −20°C overnight and then centrifuged at 13,000 rpm for 1 min. The supernatant was collected and analyzed by HPLC-MS/MS at Michigan State University.

### Statistical analysis.

Statistical comparisons were made using one‐way analysis of variance (ANOVA) using SPSS 25 software (IBM, Armonk, NY). ANOVA yielded significance (*P < *0.05); further analysis was performed using Tukey’s multiple-comparison test. The data presented were means ± standard error of mean.
