# Supplementary material for: Quorum-Sensing Master Regulator VfmE Is a c-di-GMP Effector That Controls Pectate Lyase Production in the Phytopathogen Dickeya dadantii
Source: Microbiol Spectr. 2022 Mar 30;10(2):e01805-21. doi: 10.1128/spectrum.01805-21 (PMC9045272; doi:10.1128/spectrum.01805-21)
Supplement: SUPPLEMENTAL FILE 1 — Supplemental material. Download SPECTRUM01805-21_Supp_1_seq10.pdf, PDF file, 0.3 MB [file spectrum01805-21_supp_1_seq10.pdf]

Table S1

Strains and plasmids	Relevant characteristics[a]	Reference or source
Dickeya dadantii		
3937	Wild type	(Hugouvieux-Cotte-Pattat, N.)
Δ*ecpC*	Δ*ecpC*, ABF-0020364 deletion mutant	(Yi et al, 2010)
Δ*vfmE*	Δ*vfmE*::*Km;* Km^r, ABF-0016073 deletion mutant	This study
Δ*vfmP*	Δ*vfmP*::*Km;* Km^r, ABF-0019406 deletion mutant	This study
Δ*vfmE*Δ*ecpC*	Δ*vfmE*Δ*ecpC*::*Km;* Km^r, ABF-0016073 and ABF-0020364 double deletion mutant	This study
Δ*vfmP*Δ*ecpC*	Δ*vfmP*Δ*ecpC*::*Km;* Km^r, ABF-0019406 and ABF-0020364 double deletion mutant	This study
Δ*slyA*	Δ*slyA*::*Km;* Km^r, ABF-0015312 deletion mutant	This study
Δ*vfmE*Δ*slyA*	Δ*vfmE*Δ*slyA*::*Km;* Km^r, ABF-0016073 and ABF-0015312 double deletion mutant	This study
Δ*ecpC*Δ*slyA*	Δ*ecpC*Δ*slyA*::*Km;* Km^r, ABF-0020364 deletion and ABF-0015312 double deletion mutant	This study
Δ*vfmE*Δ*ecpC*Δ*slyA*	Δ*vfmE*Δ*ecpC*Δ*slyA*::*Km;* Km^r, ABF-0016073, ABF-0020364 and ABF-0015312 triple deletion mutant	This study
Escherichia coli		
DH5α	*supE*44 Δ*lac*U169 (*ϕ*80*lacZ*ΔM15) *hsdR*17 *recA*1 *endA*1 *gyrA*96 *thi*-1 *relA*1	Lab stock
S17-1 λpir	λ(pir) *hsdR* pro *thi*; chromosomally integrated RP4-2 Tc::Mu Km::Tn7	Lab stock
NEB Express®	*fhuA2 [lon] ompT gal sulA11 R(mcr-73::miniTn10*--TetS*)2 [dcm] R(zgb-210::Tn10*--TetS*) endA1 Δ(mcrC-mrr)114::IS10*	New England Biolabs
**Plasmids**		
pKD4	Template plasmid for kanamycin cassette, Km^r	(Datsenko and Wanner, 2000)
pWM91	Sucrose-based counter-selectable plasmid, Ap^r	(Metcalf et al, 1996)
pWM91: *vfmE*	pWM91 harboring flanking regions of *vfmE* with kanamycin cassette in between, Km^r, Ap^r	This study
pWM91: *vfmP*	pWM91 harboring flanking regions of *vfmP* with kanamycin cassette in between, Km^r, Ap^r	This study
pWM91: *slyA*	pWM91 harboring flanking regions of *slyA* with kanamycin cassette in between, Km^r, Ap^r	(Zou et al, 2012)

pET21b	Overexpression and purification vector, Ap^r	Novagen
pET21b: *ycgR*$_{3937}$	Overexpression of *ycgR*$_{3937}$ in expression vector	(Yuan et.al, 2015)
pMAL-c6t	Overexpression and purification vector, Ap^r	New England Biolabs
pMAL-c6t: *vfmE*	Overexpression of *vfmE* in expression vector	This study
pMAL-c6t: *vfmE*^{R93D}	Overexpression of *vfmE*^{R93D} in expression vector	This study
pCL1920	Low copy number plasmid, lac promoter, Sp^r	(Lerner and Inouye, 1990)
pCL-*vfmE*	*vfmE* with natural promoter cloned in pCL1920, Sp^r	This study
pCL- *vfmE*^{R93D}	*vfmE*^{R93D} with natural promoter cloned in pCL1920, Sp^r	This study
pPROBE-AT	Promoter-probe vector, promoter-less *gfp*, Ap^r	(Miller et al, 2000)
pAT-*pelD*	pPROBE-AT containing *pelD* promoter-*gfp* transcriptional fusion, Ap^r	(Peng et al, 2006)
pAT-*gcpA*	pPROBE-AT containing *gcpA* promoter-*gfp* transcriptional fusion, Ap^r	From lab storage
pAT-*egcpB*	pPROBE-AT containing *egcpB* promoter-*gfp* transcriptional fusion, Ap^r	(Yi et al, 2010)
pAT-*ecpC*	pPROBE-AT containing *ecpC* promoter-*gfp* transcriptional fusion, Ap^r	(Yi et al, 2010)
pAT-*slyA*	pPROBE-AT containing *slyA* promoter-*gfp* transcriptional fusion, Ap^r	(Zou et al, 2012)

[a]Ap^r, ampicillin resistance; Km^r, kanamycin resistance; Sp^r, streptomycin resistance.

Table S2

Primers	Sequences (5'-3')	Amplicon
vfmE-A-XhoI	AATACTCGAGTCGTTTCCTGTTCATCTGC	*vfmE* deletion
vfmE-B	GAAGCAGCTCCAGCCTACACCGGTGTCATCC	
vfmE-C	CTAAGGAGGATATTCATATGTCTGTAATTAA	
vfmE-D-NotI	AATATTATGCGGCCGCTCGGCAAAATGATCGAC	
vfmP-A- XhoI	AATACTCGAGGCTGGGTTATGTGCGC	*vfmP* deletion
vfmP-B	GAAGCAGCTCCAGCCTACACCTTCTACCCGACGTCCTAGC	
vfmP-C	CTAAGGAGGATATTCATATGACCGGCCCGTTTCC	
vfmP-D- NotI	AATATTATGCGGCCGCTTCAAAGCAGATGTGGTA	
vfmE-R93D-1	TGCGCTGGATTTGGGACGTACGCCTGACGC	*vfmE* site-
vfmE-R93D-2	GCGTCAGGCGTACGTCCCAAATCCAGCGCA	directed mutant
vfmE-for-BamHI	TATCGTCGACGGATCCATGAGCTTGCAGAACACCTACG	*vfmE*
vfmE-rev-HindIII	GTTTTATTTGAAGCTTATTACAGAGGTTCTGGATATTATCCAGCAG	overexpression
vfmE-for-XbaI	AATATCTAGATGACTGGCCCTTCCGCTGA	*vfmE*
vfmE-rev-HindIII	TTATAAGCTTATACCGCCACTTATTCAGT	complementation
vfmE-p1-SalI	AATAGTCGACCTTTTTCCAGCGCCTGAATGACCCGCGC	*vfmE* promoter
vfmE-p2-EcoRI	TTATGAATTCCGTATGGGCGTAGGTGTTCTGCAAGCTC	
P1	GCGATTGTGTAGGCTGGAGCTGCTTC	Kanamycin
P2	GCTGACATGGGAATTAGCCATGGTCC	cassette
		amplification from
		pKD4 plasmid

Figure S1

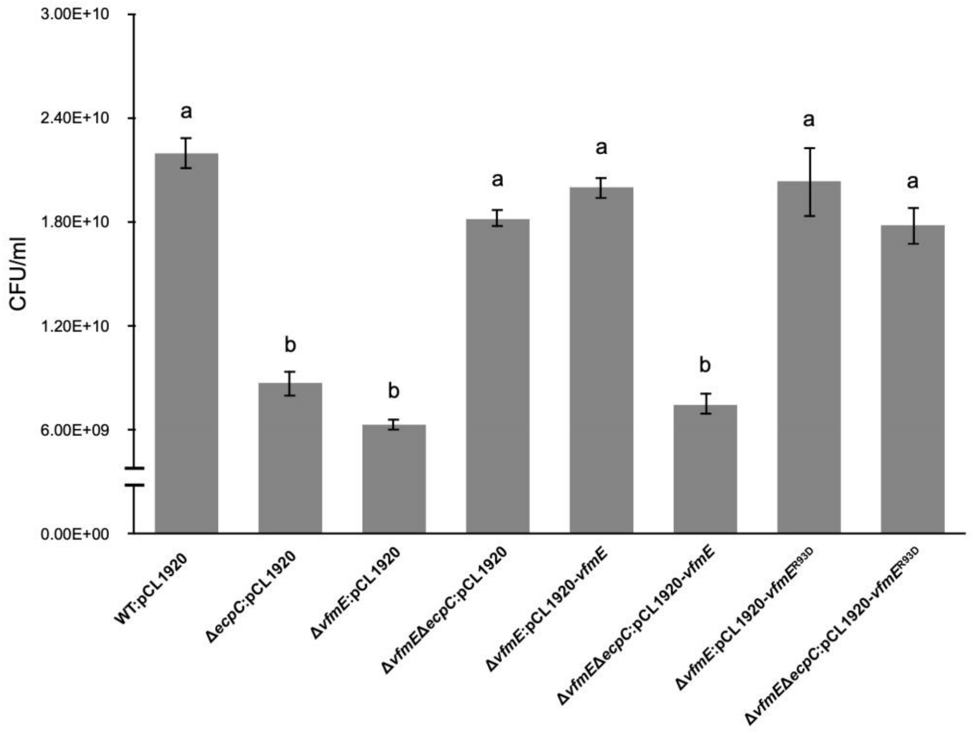

Bacterial survival rate in host

The CFU of bacterial cells was measured from the necrotic tissue fluid. Values are representative of two independent experiments, and three replicates were used for each experiment. Labels above the bars indicate statistically significant differences between treatments for 24 h ($P < 0.05$) by one-way ANOVA.

Figure S2

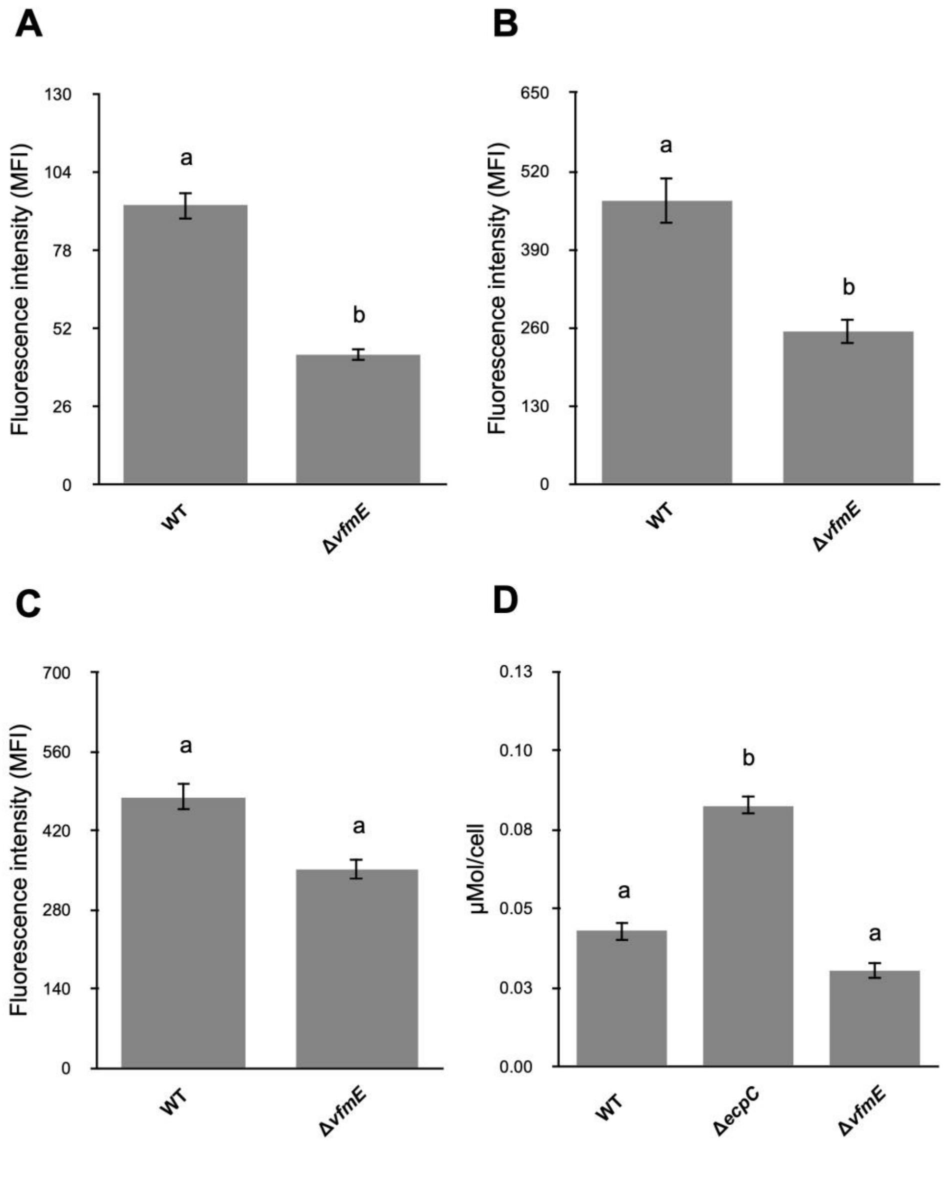

VfmE affects the transcription of c-di-GMP modulating proteins

The promoter activities of pPROBE-AT plasmids harboring *gcpA*-GFP, *egcpB*-GFP, and *ecpC*-GFP promoter regions were measured by flow cytometry. (A) *gcpA* promoter activity in wild type and Δ*vfmE* mutant. (B) *egcpB* promoter

activity in wild type and Δ*vfmE* mutant. (C) *ecpC* promoter activity in wild type and Δ*vfmE* was measured. (D) The intracellular c-di-GMP was measured by LC-MS method in wild type, Δ*ecpC,* and Δ*vfmE* mutants. Values are representative of four experiments, and three replicates were used for each experiment. Labels above the bars indicate statistically significant differences between treatments for 24 h ($P < 0.05$) by one-way ANOVA.

Values are representative of two independent experiments, and three replicates were used for each experiment. Labels above the bars indicate statistically significant differences between treatments for 12 h ($P < 0.05$) by one-way ANOVA.

References

1. Yi X, Yamazaki A, Biddle E, Zeng Q, Yang CH. Genetic analysis of two phosphodiesterases reveals cyclic diguanylate regulation of virulence factors in Dickeya dadantii. Molecular Microbiology. 2010;77(3):787-800.
2. Datsenko KA, Wanner BL. One-step inactivation of chromosomal genes in Escherichia coli K-12 using PCR products. Proceedings of the National Academy of Sciences. 2000;97(12):6640.
3. Metcalf WW, Jiang W, Daniels LL, Kim SK, Haldimann A, Wanner BL. Conditionally replicative and conjugative plasmids carrying lacZ alpha for cloning, mutagenesis, and allele replacement in bacteria. Plasmid. 1996;35(1):1-13.

4. Yuan X, Khokhani D, Wu X, Yang F, Biener G, Koestler BJ, et al. Cross-talk between a regulatory small RNA, cyclic-di-GMP signalling and flagellar regulator FlhDC for virulence and bacterial behaviours. Environmental Microbiology. 2015;17(11):4745-63.
5. Lerner CG, Inouye M. Low copy number plasmids for regulated low-level expression of cloned genes in Escherichia coli with blue/white insert screening capability. Nucleic acids research. 1990;18(15):4631.
6. Miller WG, Leveau JH, Lindow SE. Improved gfp and inaZ broad-host-range promoter-probe vectors. Mol Plant Microbe Interact. 2000;13(11):1243-50.
7. Peng Q, Yang S, Charkowski AO, Yap MN, Steeber DA, Keen NT, et al. Population behavior analysis of dspE and pelD regulation in Erwinia chrysanthemi 3937. Mol Plant Microbe Interact. 2006;19(4):451-7.
8. Zou L, Zeng Q, Lin H, Gyaneshwar P, Chen G, Yang CH. SlyA regulates type III secretion system (T3SS) genes in parallel with the T3SS master regulator HrpL in Dickeya dadantii 3937. Appl Environ Microbiol. 2012;78(8):2888-95.
